# Double Dislocation of the Superior Maxilla

**Published:** 1895-10

**Authors:** H. H. Spiers

**Affiliations:** Ravenna, O.


### Double Dislocation of the Superior Maxilla.

BY H. H. SPIERS, M.D., RAVENNA, O.

On November 20, 1894, I was called to see a double dislocation of the inferior maxilla, caused by yawning or gaping during the chill of an ordinary intermittent.

The dislocation remained unreduced for nearly four hours, owing to residence in the country and absence of a local physician.

The reduction was readily effected by placing the thumbs of the attendant far back on the body of the inferior maxilla, slightly pressing downward, at the same time using the fingers of both hands to elevate the anterior portion.

The patient, a female, aged forty-two, of slender build and of nervous temperament, had fallen down stairs two years previously and injured the spine, with imperfect recovery.

We have, then, a delicate organism previous and present injury, with malaria.

No doubt some of our distinguished contemporaries would give heroic doses of quinia sulphas and dismiss the case as cured. Or perhaps they might search for plasmodium malaria before giving anything. Either action would be entirely proper in the premises.

But with a five days' previous history of chill at 4 A.M., followed by fever and then a profuse sweat, ordinary practitioners prescribe at once.

Quinia in large doses is not well tolerated, and does little, if any, good. Muriatic acid in conjunction cannot be taken. Fowler's solution and iodide of potassium break the intermittent. A blister to the spine, a bandage to the jaw, and nature does the rest.

The patient makes a perfect recovery, and has been in good health every since.

In what way did Fowler's solution and iodide of potassium break the intermittent? Possibly by killing the plasmodium malaria. Probably by eliminating the malarial poison from the system.

